# Experimental model of mild diabetes: long-term evaluation of glycemic profile and gastric contractility in rats

**DOI:** 10.1186/1758-5996-7-S1-A10

**Published:** 2015-11-11

**Authors:** Andrieli Taise Hauschildt, Viviane Batista Behenck, Maysa Bruno de Lima, Denize Jussara Rupolo Dall'Agnol, Gustavo Tadeu Volpato, Madileine Francely Américo

**Affiliations:** 1 Universidade Federal de Mato Grosso, Barra do Garças, Brazil

## Background

Diabetes mellitus is characterized by hyperglycemia resulting from progressive defects in insulin secretion and/or insulin resistance. Diabetes has been associated with several gastrointestinal complications; however, appropriate experimental models are still under study.

## Objective

The aim was to evaluate gastric contractility at three and six months after the induction of mild diabetes in rats.

## Materials and methods

Male Wistar rats were divided into mild diabetes (n=14) and control (n=8) groups. For mild diabetes induction, newborn rats received streptozotocin (STZ-100 mg/kg, subcutaneously) in a citrate buffer and control animals received only citrate buffer on the first day of life. The oral glucose tolerance test (OGTT) was performed three months later and blood glucose was measured monthly by glucometer Fácil True Read (Home Diagnostics®). The gastric contractility was evaluated by Alternate Current Biosusceptometry (ACB), a previously validated and noninvasive biomagnetic technique. After 12 h fasting, the animals were fed with 2g of magnetically marked chow and anesthetized with sodium pentobarbital (40 mg/kg, intraperitoneally). All rats were placed in a supine position with ACB sensor positioned on the gastric region during 30 min. Frequency and amplitude of gastric contractions, also abnormal rhythmic index, were obtained from signals. Comparisons were performed by ANOVA (Tukey), being significant at p<0.05. OGTT confirmed diabetes in the STZ-induced group, demonstrating the success of the model.

## Results

Mild blood glucose levels were observed in STZ-induced group at 3rd (155.8±32.3 mg/dL) and 6th months (134.3±9.5 mg/dL) compared to control (97.0±10.9 mg/dL). There was no significant difference in frequency of gastric contraction between groups. In contrast, the amplitude of contractions were stronger in moderately diabetic rats after three (0.26±0.13 Volts/s) and six months (2.22±0.96 Volts/s) compared to control (0.018±0.07 Volts/s), and this change was more pronounced at 6th month than in the 3rd month. Abnormalities in the stomach contractions rhythm during the control (9.2±5.3%), 3rd (46.4±8.1%) and 6th months (39.0±13.3%) post-induction corroborate these motor disorders temporally associated with diabetes.

## Conclusions

Moderately elevated blood glucose levels over a long period may be related to the gastrointestinal complications since the gastric contractility is more impaired in the sixth than third month, by a mechanism that may be associated with neuropathy.

